# A Novel Homozygous *KLHL3* Mutation as a Cause of Autosomal Recessive Pseudohypoaldosteronism Type II Diagnosed Late in Life

**DOI:** 10.1159/000521626

**Published:** 2022-01-28

**Authors:** Annika Etges, Nicole Hellmig, Gudrun Walenda, Bassam G. Haddad, Jan-Philipp Machtens, Thomas Morosan, Lars Christian Rump, Ute I. Scholl

**Affiliations:** ^a^Department of Nephrology, School of Medicine, Heinrich-Heine-Universität Düsseldorf, Düsseldorf, Germany; ^b^Department of Nephrology and Medical Intensive Care, Charité, Universitätsmedizin Berlin, Corporate Member of Freie Universität Berlin and Humboldt-Universität zu Berlin, Berlin, Germany; ^c^Berlin Institute of Health at Charité, Universitätsmedizin Berlin, Center of Functional Genomics, Berlin, Germany; ^d^Institute of Biological Information Processing (IBI-1), Molekular- und Zellphysiologie, and JARA-HPC, Forschungszentrum Jülich, Jülich, Germany; ^e^Institute of Clinical Pharmacology, RWTH Aachen University, Aachen, Germany; ^f^DaVita Dialysezentrum Dormagen, Dormagen, Germany

**Keywords:** Familial hyperkalemic hypertension, Gordon's syndrome, Chronic kidney disease, Kelch-like 3, WNK4

## Abstract

**Introduction:**

Pseudohypoaldosteronism type II (PHA II) is a Mendelian disorder, featuring hyperkalemic acidosis and low plasma renin levels, typically associated with hypertension. Mutations in *WNK1*, *WNK4*, *CUL3*, and *KLHL3* cause PHA II, with dominant mutations in *WNK1*, *WNK4*, and *CUL3* and either dominant or recessive mutations in *KLHL3*. Fourteen families with recessive *KLHL3* mutations have been reported, with diagnosis at the age of 3 months to 56 years, typically in individuals with normal kidney function.

**Methods:**

We performed clinical and genetic investigations in a patient with hyperkalemic hypertension and used molecular dynamics simulations, heterologous expression in COS7 cells, and Western blotting to investigate the effect of a *KLHL3* candidate disease mutation on WNK4 protein expression.

**Results:**

The patient, a 58-year-old woman from a consanguineous family, showed hypertension, persistent hyperkalemic acidosis associated with severe muscle pain, nephrolithiasis, chronic kidney disease (CKD), and coronary heart disease. Therapy with hydrochlorothiazide corrected hyperkalemia, hypertension, and muscle pain. Genetic analysis revealed a homozygous p.Arg431Trp mutation at a highly conserved KLHL3 position. Simulations suggested reduced stability of the mutant protein, which was confirmed by Western blot. Compared with wild-type KLHL3, cotransfection of p.Arg431Trp KLHL3 led to increased WNK4 protein levels, inferred to cause increased NaCl reabsorption via the thiazide-sensitive carrier and PHA II.

**Conclusions:**

Even in patients presenting late in life and in the presence of CKD, PHA II should be suspected if renin levels are low and hyperkalemic acidosis and hypertension are inadequate for CKD stage, particularly in the presence of a suspicious family history.

## Introduction

Systemic arterial hypertension affects over 1.1 billion adults worldwide [1] and is considered the most important modifiable risk factor for all-cause morbidity and mortality worldwide [2]. Approximately 90% of adult patients have so-called primary or essential hypertension with multifactorial gene environment etiology [2], whereas an underlying cause of hypertension can be identified in the remainder. Common causes of secondary hypertension are primary aldosteronism, obstructive sleep apnea, parenchymal renal disease, and renal artery stenosis [3]. In rare cases, hypertension is inherited as a monogenic trait. Affected patients typically present with low plasma renin levels, whereas aldosterone and electrolyte levels vary depending on the underlying etiology [4]. Mendelian forms of hypertension often manifest in childhood or youth. Adults are rarely investigated for monogenic hypertension; thus, its prevalence in the general population remains unknown. In a selected cohort (inclusion criteria at least one of: age at onset ≤35 years, resistant hypertension, hypertension with electrolyte abnormalities, hormonal abnormalities, abnormal imaging results, or suggestive clinical signs), 37 candidate genes were screened, and pathogenic or likely pathogenic variants were identified in 33 of 1,179 cases (2.8%) [5].

This study focuses on pseudohypoaldosteronism type II (PHA II), a form of Mendelian hypertension that features hyperkalemic acidosis and low plasma renin levels [6]. PHA II is also known as familial hyperkalemic hypertension. It was first described in 1964 by Paver and Pauline in a young male with severe hypertension and hyperkalemia despite otherwise normal renal function [7] who was further characterized by Stokes and colleagues [8] and Arnold and Healy [9] and shown to have low plasma renin. Following the report of a 10-year-old girl with short stature, hypertension, severe hyperkalemia, mild acidemia, periodic paralysis, and suppressed renin in 1970 by Gordon et al. [10], the syndrome has also occasionally been referred to as Gordon's syndrome.

**Fig. 1:**
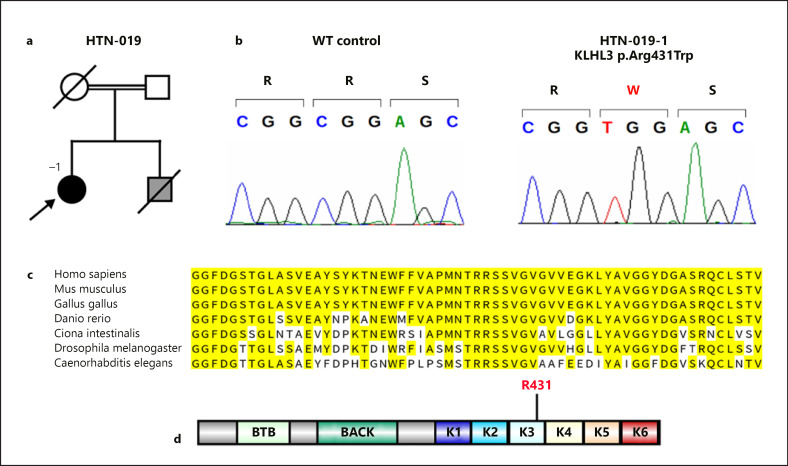
**a** Pedigree of kindred HTN-019. Arrow points to the index case. Female subjects are shown as circles, and male subjects as squares. Double line indicates consanguinity. Slashes through symbols indicate deceased individuals. Black symbol, affected; white symbol, unaffected; gray symbol: status unknown. **b** Sanger sequences of HTN-019-1 and of an unrelated WT control. Encoded amino acids are shown in one-letter code above, with the mutant amino acid in red. **c** Alignment of orthologs in different species (top) demonstrates conservation of Arg431. **d** Location of the mutant residue in a cartoon of human KLHL3, with Kelch-like repeats (K1–K6) displayed in different colors. Also shown are BTB and BACK domains (see text).

In 2001, mutations in the with-no-lysine (WNK) serine threonine kinase genes *WNK1* and *WNK4* were identified as causes of PHA II [11], and further mutations in the *KLHL3* (encoding kelch-like 3) and *CUL3* (encoding cullin 3) genes were described in 2012 [12, 13]. PHA II subforms caused by mutations in *WNK1*, *WNK4*, and *CUL3* are autosomal-dominant disorders, whereas *KLHL3* mutations can be inherited either in an autosomal-dominant or in an autosomal-recessive fashion [12]. KLHL3 features an N-terminal BTB domain, followed by a BACK domain and a C-terminal six-bladed β-propeller structure consisting of so-called kelch-like repeats (shown in Fig. 1d, 2a). Dominant KLHL3 mutations cluster in or between propeller blades, whereas recessive mutations are distributed throughout the protein [12]. Recessive cases are less common than dominant cases; in total, 21 patients from 14 families with recessive *KLHL3* mutations have been published [12, 13, 14, 15] (Table 1).

The underlying pathophysiology of hypertension in PHA II is increased NaCl reabsorption via the thiazide-sensitive carrier (Na-Cl cotransporter, NCCT) in the distal convoluted tubule of the kidney, and therapy with thiazides corrects both hypertension and electrolyte abnormalities in patients with PHA II [16]. In short, WNK1 and WNK4 phosphorylate OSR1 (oxidative stress-responsive gene 1) and SPAK (Ste20-related proline-alanine-rich kinase), which then phosphorylate and activate NCCT [17, 18]. KLHL3 is a substrate adapter of the ubiquitin ligase CUL3; the KLHL3-CUL3 complex ubiquitinylates WNK kinases and thus promotes their degradation [19]. Loss of ubiquitin ligase function or impaired binding to WNK kinases causes increased WNK abundance, increased NCCT phosphorylation [18], and increased NaCl absorption in the distal convoluted tubule. In later segments of the nephron, NaCl reabsorption via ENaC (epithelial sodium channel) and K^+^ secretion via ROMK (renal outer medullary potassium channel) are reduced [20, 21]. *KLHL3* knockout mice show hypoplasia of the distal convoluted tubule, strongly increased levels of WNK1 and WNK4, and increased OSR1, SPAK, and NCC phosphorylation in the kidney. Further, they display hyperkalemia, hyperchloremia, and metabolic acidosis [22]. Here, we report a patient with autosomal-recessive PHA II and characterize the underlying genetic mutation and pathophysiology.

## Materials and Methods

### DNA Preparation and Sanger Sequencing

DNA was prepared from a peripheral venous blood sample using the QIAamp DNA Blood Maxi Kit (Qiagen) according to the manufacturer's instructions. Standard PCR and direct bidirectional Sanger sequencing of genomic DNA were performed using published primers for *KLHL3* [13], *WNK1*, and *WNK4* [11] and the CUL3_9F (5′-AGGAGACACTTTCTCAAACCG-3′) and CUL3_9R (5′-TGTTCTTCTCCAAAACAATCTACC-3′) primers for *CUL3*. Sanger sequencing was performed at Eurofins Genomics.

### Sequence Alignment

Homologous protein sequences were identified using NCBI Protein BLAST (https://blast.ncbi.nlm.nih.gov/Blast.cgi) and the eggNOG database (http://eggnogdb.embl.de). Sequences shown include human KLHL3 and its orthologs: *Homo sapiens* (NP_059111.2), *Mus musculus* (NP_001349344.2), *Gallus gallus* (XP_015149442.1), *Danio rerio* (XP_021328460.1), *Ciona intestinalis* (XP_009862126.1), *Drosophila melanogaster* (NP_724095.1), and *Caenorhabditis elegans* (NP_001254310.1). Human paralogs were identified by literature research and include KLHL1 to KLHL42 as shown in online supplementary Table 1 (for all online suppl. material, see www.karger.com/doi/10.1159/000521626) [23]. Sequences were aligned using Jalview version 2.10.5 [24]. Domain structure was from UniProt (Q9UH77, accessed on April 13, 2021) and was visualized using DOG [25].

### Plasmids and Site-Directed Mutagenesis

The pTRE2hyg WNK4 and pFN21A KLHL3 plasmids were kind gifts of Dr. Shinichi Uchida (Tokyo Medical and Dental University). To introduce the p.R431W mutation, primers were designed using Primer X (https://www.bioinformatics.org/primerx): 5′- GATGAACACGCGG**T**GGAGCAGTGTGG −3′ (KLHL3_R431W_1F) and 5′- CCACACTGCTCC**A**CCGCGTGTTCATC −3′ (KLHL3_R431W_1R). Mutant residues are shown in bold. Site-directed mutagenesis was performed using the QuikChange Site-Directed Mutagenesis Kit with a PfuUltra High-Fidelity DNA Polymerase (Agilent Technologies, Santa Clara, CA, USA) according to the manufacturer's instructions. The cDNA was Sanger-sequenced at Eurofins Genomics. Plasmids were prepared using the QIAGEN Plasmid Plus Maxi Kit.

### Tissue Culture, Transient Transfection, and Western Blot

COS7 cells were cultured in DMEM + L-glutamine, 10% FBS, and 1% penicillin/streptomycin (all Gibco, Thermo Fisher). For transfections, cells were seeded on 6-well plates at a density of 1.9 × 10^5^ cells/well and grown to approximately 90% density. Cells were transfected with 1 µg pTRE2hyg WNK4 and 2 µg pFN21A (empty vector or KLHL3 wild type [WT]) or KLHL3 R431W cDNA using Lipofectamine 2000 (Thermo Fisher Scientific) according to the manufacturer's instructions. Two independent clones each were used for transfection. Forty-eight hours after transfection, cells were washed in cold PBS and lysed in 10 mM Tris-HCl (pH 7.5), 150 mM NaCl, 1 mM EDTA, and 1% NP40 containing cOmplete Protease Inhibitor Cocktail (Roche, Merck). The lysate was centrifuged at 20,000 g for 30 min at 4°C, and the supernatant was stored at −20°C. Protein concentrations were determined in duplicates (standard) or triplicates (samples) by the BCA assay (Pierce, Thermo Fisher) according to the manufacturer's instructions. About 20 µg total protein was fractionated by SDS-PAGE and transferred to a PVDF membrane (1.5 h, 100 V). Membranes were blocked in 2% dry milk in TBST. Western blot was performed using polyclonal rabbit anti-HaloTag (Promega #G9281, 1:500, 4°C overnight), followed by washing and incubation with donkey IgG anti-rabbit IgG (H + L)-HRPO (Jackson ImmunoResearch # 711-035-152, dianova, 1:20,000, 1 h at room temperature), washing, and enhanced chemiluminescent detection (Amersham ECL Prime Western Blotting Detection Reagent, GE Healthcare). Blots were stripped using ROTIFree Stripping Buffer 2.0 (Carl Roth), blocked in 2% dry milk in TBST overnight at 4°C, and incubated with monoclonal mouse anti-FLAG M2 (Sigma-Aldrich #F1804, Merck, 1:1,000, 1.5 h at room temperature), followed by donkey IgG anti-mouse IgG (H + L)-HRPO (Jackson ImmunoResearch # 715-035-151, dianova, 1:20,000, 1 h at room temperature), ECL detection, stripping, and blocking as above. Blots were then incubated with monoclonal mouse anti-β-actin (Sigma-Aldrich # A2228, Merck, 1:5,000, 1 h at room temperature) and donkey IgG anti-mouse IgG, again followed by ECL detection. Bands were quantified using the Image Lab Software on a ChemiDoc XRS+ (Bio-Rad). Intensities of bands detected by anti-HaloTag antibody and anti-FLAG M2 antibody, respectively, were divided by corresponding intensities of bands detected by anti-β-actin antibodies. To assess expression levels of KLHL3, cells were transfected as above however by using 0 µg, 0.5 µg, 1 µg, 2 µg, 4 µg, and 8 µg of pFN21A KLHL3 WT or R431W. Gel electrophoresis and blotting were performed as above. For the cycloheximide chase assay, cells were seeded as above and transfected as above however with 2 µg pFN21A KLHL3 WT or 4 µg KLHL3 R431W. Forty-eight hours after transfection, 100 µM cycloheximide was added, and cells were lysed after the indicated time points. Further analysis steps were as above.

### Molecular Dynamics Simulation

The WT model was based on the X-ray crystal structure (PDBID: 4CH9) of the human Kelch domain in complex with the WNK4 peptide-fragment [26]. Missing atoms and terminal caps were added using the *psfgen* utility in visual molecular dynamics version 1.9.3 [27]. The mutant (R431W) KLHL3 model was generated using Modeller version 9.18 [28]. The final residue range for each system was 300–585. The N- and C-termini of the WT and R431W models were neutralized by acetylation and N-methylamidation, respectively. Default protonation states were assigned to each titratable residue according to analysis with PROPKA version 3.0 [29]. The WNK4 peptide was removed from production simulations due to dissociation from KLHL3 after initial equilibration simulations.

All simulations were conducted using the GROMACS software package version 2021 [30]. CHARMM36m force-field parameters were used for protein atoms [31]. Ions were described using default CHARMM parameters, and the TIP3P model was used for waters [32]. An integration time step of 1 fs was used for the initial equilibration step, with 2 fs being used for all subsequent simulations. All bonds involving hydrogen were constrained using LINCS [33]. Nonbonded Van der Waals interactions were calculated using the Lennard-Jones potential with a cutoff radius of 1.2 nm; forces were smoothly switched off in the range of 1.2–1.0 nm. All electrostatics were calculated using the smoothed particle-mesh Ewald [34] method with a real-space cutoff distance of 1.2 nm. Following an initial simulation in the isochoric-isothermal ensemble (NVT), all subsequent simulations were performed in the isobaric-isothermal ensemble (NPT) at a temperature of 310.15 K using the velocity-rescaling thermostat [35] with a time constant of 0.5 ps. The thermostat was applied separately to the protein and solvent (i.e., water and ions); the same groups were used for the removal of center-of-mass (COM) motion. A pressure of 1 bar was imposed using either a Berendsen [36] or Parrinello-Rahman [37] barostat in an isotropic manner with a time constant of 5 ps.

The WT and R431W models were solvated in a cubic box with initial dimensions of 1.2 × 1.2 × 1.2 nm^3^. Each system was neutralized with 150 mM NaCl. Following an initial steepest-descent energy minimization, each system was equilibrated in the NVT ensemble for 5 ns with position restraints on all protein atoms. A subsequent equilibration step was performed in the NPT ensemble for 5 ns with the same restraints as in the previous step. Position restraints were removed from all side chains for the final 5-ns equilibration simulation. Three initial structures were generated from the final stage of equilibration for independent production simulations of the WT and R431W systems with all position restraints removed. Each production replicate was simulated for 1.1 µs; the first 0.1 µs was removed from analysis.

To assess the structural stability over the course of the simulations, root mean squared deviations (RMSDs) from the crystal structure were calculated for the Cα atoms of the entire protein, as well as the K3–K4 interface (residues 425–465) and the WNK4-binding pocket (residues 339, 355, 360, 386, 402, 407, 432, 449, 451, 481, 498, 528, and 577). Running averages of the RMSD traces were calculated and overlaid with the raw data.

The effect of the mutation on structural stability was visualized by calculating the difference in the mean square fluctuations of the protein Cα atoms, averaged over frames and replicas and mapping to the WT protein structure. All protein visualizations were generated using ChimeraX [38]. The effect of the R431W mutation on the K3 (residues 425–441) to K4 (residues 442–465) interface was characterized by hydrogen bond (H-bond) analysis and COM distance between the two groups. H-bonds were assessed at each frame using the GROMACS tool *gmx hbond*, with distance and angle cutoffs of 3.5 Å and 30°, respectively. COM distances were similarly assessed at each frame − distributions of both the H-bonds and COM distances were visualized as violin plots. Averages were calculated over trajectories and replicas.

Electrostatics of the WNK4-binding pocket was calculated using *g_elpot* [39]. Time course of the electrostatic potential in the binding pocket was evaluated by defining a spherical volume with a radius of 8 Å, centered at the geometric center of the residues comprising the binding pocket. To evaluate the variance in the electrostatics, 50-ns block averages were calculated. The distribution of the electrostatic potential within the binding pocket for the WT and R431W mutant was visualized as violin plots, with averages calculated over 50-ns blocks and trajectories.

## Results

### Case Report

The subject, a female of Turkish origin, was referred at the age of 58 years to the outpatient clinic of the Department of Nephrology, University Hospital Düsseldorf, for persistent hyperkalemia (first diagnosed at age 47 years), suspected type IV renal tubular acidosis in the presence of chronic kidney disease (CKD) (G3a A2, KDIGO), cirrhosis of the left kidney, nephrolithiasis with a history of surgical stone removal at age 17 years, recurrent urinary tract infections, and hypertension (first diagnosed at the age of 41 years). Her past medical history was significant for coronary heart disease, with status post-non-ST segment elevation myocardial infarction at the age of 47 years, multiple stent implantations, and ST elevation myocardial infarction 5 months prior to referral. After the second myocardial infarction, serum potassium levels had risen up to 7.3 mmol/L, and severe muscle pain had started. The patient also reported paroxysmal atrial fibrillation, hypercholesterolemia, and allergy to contrast agents. Medications included bisoprolol 2.5 mg bid, calcium polystyrene sulfonate 15 g bid, sodium hydrogen carbonate 3 g tid, phenprocoumon, atorvastatin 40 mg qd, aspirin 100 mg qd, pantoprazole 40 mg qd, L-thyroxin 25 μg qd, and clopidogrel 75 mg qd. Temporary discontinuation of statin therapy had not alleviated muscle pain. Discontinuation of an AT1 receptor antagonist that had been on her regimen as well as a diet low in potassium had not led to an improvement of hyperkalemia.

Family history was significant for parental consanguinity (I° cousins), hypertension in both parents, a fatal stroke in her mother in her early 60s, and coronary artery disease in her father. Her brother had died from renal disease of unclear origin in Turkey in his 50s (shown in Fig. 1 a).

At the time of evaluation, blood pressure was 148/87 mm Hg, and her heart rate was 60/min. Clinical examination was unremarkable, apart from mild edema of the lower extremities, multiple hematomas, and striae distensae.

Laboratory examination showed hyperkalemia (serum potassium of 5.3 mmol/L [normal 3.6–4.8]), normal serum sodium (139 mmol/L, normal 135–145), hyperchloremia (107 mmol/L, normal 95–105), hypomagnesemia at 0.55 mmol/L (normal 0.73–1.00), elevated serum creatinine at 1.18 mg/dL (normal <0.90) with an estimated glomerular filtration rate of 51 mL/min (CKD-EPI [40], normal 90–140), elevated urea of 47 mg/dL (normal 21–43), suppressed renin concentration (<1.0 pg/mL [normal 1.7–23.9]), and aldosterone of 157 pg/mL (normal 12–236). Serum calcium and phosphate were normal (2.31 mmol/L [normal 2.10–2.42] and 0.95 mmol/L [normal 0.84–1.45], respectively). Venous blood gas analysis revealed pH 7.33 (normal 7.35–7.43), standard bicarbonate 19.8 mmol/L (normal 24–30), and standard base excess −5.3 mmol/L (normal −2 to +3). Spot urinary potassium was low (6.5 mmol/L [normal 10–200]), whereas spot urinary calcium was normal (0.36 mmol/L); urinary creatinine was 24.2 mg/dL. There was no detectable albuminuria or proteinuria.

Renal ultrasound demonstrated a small right kidney (8.5 cm in length) with thin, inhomogeneous parenchyma, suspected scarring, and a 12-mm well-defined round cyst. The left kidney was normal in size (10 cm in length), with normal parenchymal thickness, a 22-mm well-defined round cyst, and a complicated 12-mm well-defined round cyst with a suspected septum. There was no evidence of hydronephrosis.

The presence of hyperkalemic metabolic acidosis with inadequately low urinary potassium excretion, which was not explained by CKD alone, low-renin hypertension with aldosterone that was inadequately low in for hyperkalemia, and parental consanguinity led us to consider PHA II. Initiation of therapy with hydrochlorothiazide 25 mg qd led to a decrease of serum potassium to 4.2 mmol/L within 1 week, and blood pressure normalized. Calcium polystyrene sulfonate and sodium hydrogen carbonate were rapidly discontinued. Temporary reduction of hydrochlorothiazide to 12.5 mg qd led to the reappearance of hyperkalemic acidosis. Hyponatremia occurred after hydrochlorothiazide therapy and resolved upon a diet rich in salt. Two months after the first visit to our clinic, the initially reported muscle pain had resolved. Minor pain that was described as different in character was attributed to statin therapy in the presence of mild CK (153 U/L, normal <145) and LDH (250 U/L, normal <147) elevation. Review of prior therapy for hypertension revealed a regimen of valsartan plus hydrochlorothiazide (12.5 mg) prior to myocardial infarction, after which hydrochlorothiazide had been discontinued, apparently unmasking electrolyte abnormalities and causing muscle pain.

### Genetic Analysis

Because of the clinical suspicion of PHA II, we performed PCR and Sanger sequencing of the *KLHL3*, *CUL3*, *WNK1*, and *WNK4* genes (see Methods). No variants in the *WNK1*, *WNK4*, and *CUL3* genes were identified. However, a homozygous chr5:g.136973013 G > A (GRCh37) variant was identified in the *KLHL3* gene, resulting in a p.Arg431Trp missense mutation (NP_059111.2) predicted as probably damaging by PolyPhen [41], deleterious by SIFT [42] and disease-causing by MutationTaster [43] (shown in Fig. 1b). This variant is present once in a heterozygous state among 251,436 alleles in gnomAD (allele frequency 4 × 10^−6^) [44] and once in a heterozygous state among 21,366 alleles in the ALFA Project (www.ncbi.nlm.nih.gov/snp/docs/gsr/alfa/; allele frequency 5 × 10^−5^). It is listed in dbSNP as rs769995865 and has not been reported in ClinVar. Arginine at human position 431 is completely conserved among 100 vertebrates (http://genome.ucsc.edu/cgi-bin/hgTrackUi?db=hg19&g=multiz100way, with the exception of horse, for which no sequence is available), in *C. intestinalis* (vase tunicate), *D. melanogaster* (fruitfly), and *C. elegans* (nematode worm) (shown in Fig. 1c). Out of 41 paralogous proteins, arginine is conserved in 17, with none of the paralogs showing tryptophan at this position (online suppl. Fig. 1). The finding of a homozygous variant in the setting of consanguinity, in combination with its rarity in the general population and the evolutionary conservation of the affected residue, led us to consider the KLHL3 p.Arg431Trp mutation as a candidate for disease causation. The variant is located in the Kelch-like repeat 3 (shown in Fig. 1d). A homozygous p.Arg431Gln variant at the identical residue has been previously described in a single individual [12] (Table 1).

### Structural Analysis

**Fig. 2:**
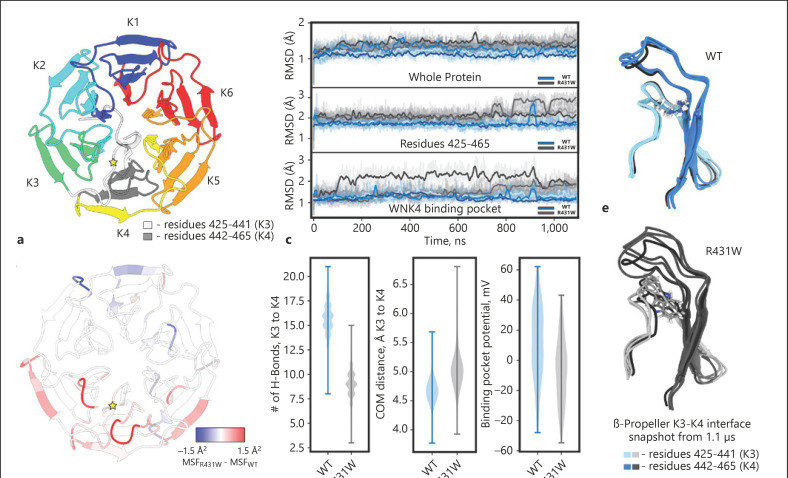
MD simulations of the WT and p.Arg431Trp (R431W) KLHL3 Kelch domains. **a** Top-down view of Kelch domain (PDBID: 4CH9), colored by β-propeller blades (K1–K6). Mutation site indicated by yellow star, and region of interest colored gray (residues 425–465), and the WNK4 peptide (not included in the simulations) transparent and colored in gray. **b** Difference in the MSFs of the Kelch domain mapped on the protein backbone. Mutation site indicated by a yellow star. Red color indicates more dynamic (less stable) parts of the protein. **c** RMSD of the whole protein (top), residues 425–465 (middle), and the WNK4-binding pocket (bottom) from the crystal structure for three independent simulation replicas (bold lines, running averages). **d** Violin plots of h-bonds (left) and COM distance (middle) between K3 (residues 425–441) and K4 (442–465) β-propeller blades and binding-pocket electrostatics (right). **e** Structural overlay of the K3–K4 interface from the final frame (1.1 μs) of each replica (WT, blue; R431W, gray) with the experimental/initial models (black). Protein backbone displayed as cartoon, with residue 431 displayed as sticks. MSF, mean square fluctuation.

The Kelch domain (shown in Fig. 2a) is composed of six “propeller blades” stabilized by inter-blade hydrogen bonds [45]. Previous simulation studies of Kelch-domain surface mutations demonstrated decreased interaction with the WNK4 peptide but found little disruption of Kelch-WNK4 binding by mutations in the core of the protein [46]. Here, we used equilibrium molecular dynamics simulations to determine the structural and dynamic consequences of the R431W core mutation. R431W is positioned at the interface between the third (K3) and fourth (K4) propeller blades, situated directly underneath the WNK4-binding site (shown in Fig. 2a). Calculation of the difference in mean squared fluctuations (a measure of variance in the protein structure) between R431W and WT simulations reproducibly demonstrated that the perturbation in protein dynamics caused by the mutation propagates throughout the entire protein, with strongly increased structural dynamics at the K3–K4 interface and the surrounding K3–K5 region, but also decreased dynamics at more distant sites (shown in Fig. 2b). Global RMSD calculations indicated a slight increase in structural instability of the mutant over the 1.1-µs simulations − focused RMSD calculations of the K3–K4 interface (residues 425–465) and the WNK4-binding site (residues 339, 355, 360, 386, 402, 407, 432, 449, 451, 481, 498, 528, and 577), suggesting a disruption of tertiary structure in the R431W mutant (shown in Fig. 2c, e; see below).

For further quantification of the interaction between the K3 (residues 425–441) and K4 (residues 442–465) blades, the number of hydrogen bonds (h-bonds) and the COM distance between the two groups were calculated. The WT interface maintains, on average, seven more h-bonds than in the R431W mutant. R431 itself only participates in 4–5 h-bonds − accounting for ∼27% of the K3–K4 h-bonds − indicating that the R431W mutation disrupts the entire interface (shown in Fig. 2d, e). Furthermore, the R431W mutant displays an increased COM distance between the two sub-domains (shown in Fig. 2d), corroborating the h-bond analysis.

To assess the effects of the mutation to WNK4 binding, we calculated the electrostatics of binding pocket throughout the 1.1-µs simulation. The R431W mutation induces a moderate change in the binding-pocket electrostatics toward negative voltages (shown in Fig. 2d). Though modest, the decreased positive potential in the binding pocket would disfavor the association of the negatively charged WNK4 acidic motif.

### Functional Analysis

**Fig. 3:**
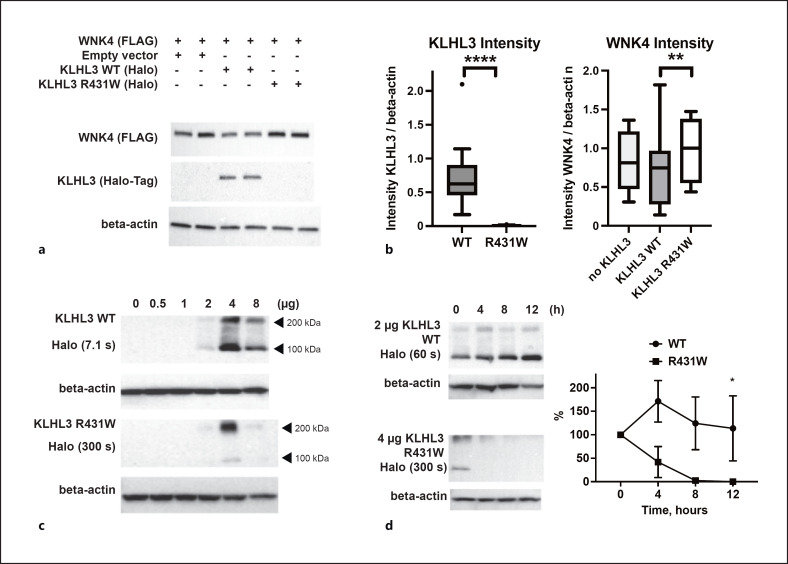
**a** Representative Western blots of cell lysates transfected with WNK4 and empty vector, WT or p.Arg431Trp (R431W) KLHL3. **b** Box and whisker plots (Tukey) with quantitation of the results from 12 experiments, using two independent clones of each plasmid. **, *p* = 0.0046; ****, *p* < 0.0001 (two-tailed ratio-paired *t* tests). **c** Representative Western blots of cell lysates transfected with increasing amounts of WT or R431W KLHL3. Note the different exposure times for WT and R431W. The lower and upper HaloTag-positive bands may reflect the KLHL3 monomer and dimer, respectively. **d** Representative Western blots of cells transfected with 2 µg WT or 4 µg R431W KLHL3 plasmid, treated with cycloheximide, and lysed at the indicated time points. Note the different exposure times. Analysis of 2 biological replicates with 2 technical replicates each shows decreased stability of mutant protein, normalized to beta-actin and time point 0 h (mean ± SD). At 12 h, significantly less R431W than WT protein is present (Mann-Whitney test, *, *p* = 0.029).

To further study the pathophysiology associated with KLHL3 p.Arg431Trp, we obtained plasmids encoding 3x FLAG-tagged human WNK4 in the pTRE2hyg vector and Halo-tagged human KLHL3 in the pFN21A vector [47]. We introduced the p.Arg431Trp mutation to the WT KLHL3 plasmid using site-directed mutagenesis and heterologously expressed the corresponding plasmids in COS7 cells. When coexpressing WT and mutant KLHL3 with WT WNK4, Western blots with anti-Halotag primary antibodies revealed WT KLHL3 bands. However, repeated transfections of two independent KLHL3 p.Arg431Trp clones initially did not result in detectable bands (relative intensity normalized by β-actin expression, 0.761 ± 0.144 in WT vs. 0.009 ± 0.002 in p.Arg431Trp, *p* < 0.0001, *t* = 12.81). Expression levels of WNK4, determined by anti-FLAG Western blot, were significantly higher in cells coexpressing KLHL3 p.Arg431Trp than in cells coexpressing WT KLHL3 (relative intensity normalized by β-actin expression, 0.721 ± 0.137 with WT vs. 0.967 ± 0.115 with Arg431Trp, *p* = 0.005, *t* = 3.546). All values are given as mean ± SEM, and comparison was performed by a two-tailed ratio-paired *t* test, *df* = 11 (shown in Fig. 3a, b). To assess whether KLHL3 p.Arg431Trp was expressed at lower quantities, we transfected increasing amounts (0, 0.5, 1, 2, 4, and 8 µg) of WT and mutant plasmids. With 8 µg, we observed increased lethality (data not shown), but with expression of 4 µg mutant plasmid, we could detect a weak band at the expected size (note the longer exposure time compared to WT, shown in Fig. 3c). To assess whether decreased protein stability could cause decreased expression levels of the mutant protein, we performed cycloheximide chase assays. KLHL3 p.Arg431Trp was degraded more rapidly than WT (shown in Fig. 3d).

## Discussion/Conclusion

At the age of 58 years, the patient reported here was diagnosed later in life than any of the previously reported cases with recessive *KLHL3* mutation. The oldest patient reported in the literature was diagnosed at the age of 56 years [15]. However, retrospectively, hypertension and hyperkalemia had been present in our patient for several years. The characteristic hyperkalemic metabolic acidosis associated with PHA II had likely been masked by administration of a thiazide diuretic. Discontinuation of hydrochlorothiazide after a myocardial infarction was the likely cause of massive hyperkalemic acidosis associated with muscle pain and hypertension that eventually led to the diagnosis of PHA II. We thus suggest that the presence of hyperkalemic metabolic acidosis that is inadequate for kidney function in combination with suppressed renin should raise the suspicion of PHA II even in patients over 50 years of age. The diagnosis of PHA II can be complicated by the effects of preexisting treatment with antihypertensive medications that influence the renin angiotensin aldosterone system and may lead to spuriously elevated or low renin and/or aldosterone values [48, 49]. Hyperkalemia despite a normal glomerular filtration rate is often described as a characteristic feature of PHA II [6]. While this may be characteristic in children, in adults, such as in the patient reported here, longstanding hypertension, nephrolithiasis, and recurrent urinary tract infections can lead to impaired kidney function, as previously reported in a patient with a heterozygous mutation [14]. Nephrolithiasis as in our patient has been described in PHA II [50] but is not a common finding. Interestingly, spot urinary calcium:creatinine ratios were not elevated in our patient (0.17 and <0.06 mmol/mmol creatinine, respectively), contrary to observations in large kindred with the *WNK4* mutation and average urinary calcium of 0.85 ± 0.27 mmol/mmol creatinine [51]. Severe target-organ damage in our patient, including myocardial infarctions and CKD, emphasize the need for early diagnosis and adequate therapy of PHA II, although hypertension and target-organ damage in our patient's parents may also point to a genetic risk independent of the homozygous PHA II mutation. To prevent delayed diagnosis, after more common causes, such as primary aldosteronism, have been excluded, genetic testing for Mendelian forms of hypertension should be considered by practitioners when patients present with low-renin hypertension in the presence of hormonal and/or electrolyte abnormalities.

Samples from the patient's family were not available for analysis; however, in the setting of consanguinity, it is highly likely that both parents were heterozygous carriers of the p.Arg431Trp mutation. Detailed information regarding the patient's deceased brother was not available. It is conceivable that he, too, suffered from PHA II, resulting in CKD that was eventually fatal.

It is possible that the p.Arg431Trp mutation observed in our patient prevents the Kelch domain from folding, consistent with the decreased structural stability of the mutated domain observed in our simulations, where the side-chain substitution was inserted in the already folded protein. Accordingly, Western blot analysis indicated significantly reduced expression levels of p.Arg431Trp KLHL3 (shown in Fig. 3a-c). The cycloheximide (an inhibitor of protein biosynthesis) chase assay further demonstrated reduced protein stability of p.Arg431Trp KLHL3 (shown in Fig. 3d), similar to prior observations in the p.Ser410Leu variant [52]. These data indicate that the primary consequence of the p.Arg431Trp mutation is to destabilize the folded state of the protein, whereas decreased interactions of folded KLHL3 with WNK4 is, at most, a secondary effect. In any case, lower levels of KLHL3 reduce WNK ubiquitinylation, resulting in higher WNK4 abundance (shown in Fig. 3) and are inferred to cause higher NCCT phosphorylation, as well as lower potassium secretion via ROMK, explaining the underlying pathophysiology.

## Statement of Ethics

The research protocol was approved by the Ethics Committee of the Medical Faculty, Heinrich Heine University Düsseldorf (study number 4330), and written informed consent in compliance with the Helsinki Declaration was obtained from the research participant for the research study and publication of this case report.

## Conflict of Interest Statement

A.E., N.H., B.G.H., J.P.M., and T.M. have no conflicts of interest to declare. L.C.R. received personal fees for consultancies and speaking engagements from Astellas, Baxter, Bayer-Vital, Boehringer, Fresenius, Medtronic, Novartis, and Recor, with no relevance to the paper. U.I.S. is listed as an inventor on patents US 10,696,739 and 16/614,401, with no relevance to the paper.

## Funding Sources

This study was funded by the Ministerium für Kultur und Wissenschaft der Landes Nordrhein-Westfalen (Rückkehrprogramm), the Stiftung Charité (BIH_PRO_406), and the German Research Foundation (DFG, SCHO 1386/2-1; project-ID 431984000, CRC 1453), all to U.I.S. It was funded by the Deutsche Forschungsgemeinschaft (German Research Foundation) to J.P.M. (MA 7525/2-1, as part of the research unit FOR 5046, project P2) and by a grant from the Interdisciplinary Centre for Clinical Research within the faculty of Medicine at the RWTH Aachen University (IZKF TN1-3/IA532003). The authors gratefully acknowledge the computing time granted through JARA on the supercomputer JURECA at Forschungszentrum Jülich. Funding agencies had no role in the preparation of the manuscript.

## Author Contributions

A.E. performed DNA preparation and sequencing, sequence alignment and mutagenesis, analyzed data, and revised the manuscript. N.H. and G.W. performed tissue culture and Western blotting. T.M., L.C.R., and U.I.S. evaluated the subject. B.G.H. produced and analyzed simulation data. J.P.M. advised on the design and analysis of molecular simulations. U.I.S. made the clinical diagnosis, recruited the subject for the study, planned and oversaw all experiments, analyzed data, and wrote the initial draft of the manuscript, with all authors contributing to a final version.

## Data Availability Statement

Clinical data that support the findings of this study are not publicly available due to patient confidentiality, but additional details can be obtained from the corresponding author upon request. Laboratory data are available on Zenodo (https://zenodo.org/record/4807754).

## Supplementary Material

Supplementary data

## Figures and Tables

**Table 1:** Overview of PHA II cases with recessive *KLHL3* mutations

Patient	*KLHL3* variants	Age at diagnosis, years	K^+^ at diagnosis, mmol/L	BP at diagnosis, mm Hg	Reference
K20-1	W470X/W470X	N/A	N/A	N/A	[12]
K20-2	W470X/W470X	N/A	N/A	N/A	[12]
K20-3	W470X/W470X	N/A	N/A	N/A	[12]
K35-2	R240X/R336I	N/A	N/A	N/A	[12]
K35-5	R240X/R336I	N/A	N/A	N/A	[12]
K35-6	R240X/R336I	N/A	N/A	N/A	[12]
K37-1	Del. C, L241 fs/Del. C, L241 fs	N/A	N/A	N/A	[12]
K53-1	R431Q/R431Q	N/A	N/A	N/A	[12]
K30-1	F322C/S410L	N/A	N/A	N/A	[12]
K30-2	F322C/S410L	N/A	N/A	N/A	[12]
K73-1	Q144X/M427T	N/A	N/A	N/A	[12]
K73-2	Q144X/M427T	N/A	N/A	N/A	[12]
K33-1	Splice donor g (+1)a/T575W	N/A	N/A	N/A	[12]
K55-1	S410L/Y557C	N/A	N/A	N/A	[12]
13-II:1	R384W/R384W	17	6.3	160/110	[13]
37-1:1	R228G/R228 G	0.25	7.3	N/A	[13]
51-I:1	P426L/P426L	16	4.7	130/71	[13]
54-I:1	P426L/P426L	7	5.7	140/90	[13]
VI-2	S553L/S553L	34	5.2	158/107	[14]
VI-4	S553L/S553L	26	5.3	115/70	[14]
N/A	T110A/T110A	56	5.4	162/80 (on medication)	[15]
HTN-019-01	R431W/R431W	58	5.3 (on medication)	148/87 (on medication)	This article

K+, serum potassium; BP, blood pressure.

## References

[1] Collaboration NCDRF (2017). Worldwide trends in blood pressure from 1975 to 2015: a pooled analysis of 1479 population-based measurement studies with 19.1 million participants. Lancet.

[2] Oparil S, Acelajado MC, Bakris GL, Berlowitz DR, Cifkova R, Dominiczak AF (2018). Hypertension. Nat Rev Dis Primers.

[3] Rimoldi SF, Scherrer U, Messerli FH (2014). Secondary arterial hypertension: when, who, and how to screen?. Eur Heart J.

[4] Athimulam S, Lazik N, Bancos I (2019). Low-renin hypertension. Endocrinol Metab Clin North Am.

[5] Bao M, Li P, Li Q, Chen H, Zhong Y, Li S (2020). Genetic screening for monogenic hypertension in hypertensive individuals in a clinical setting. J Med Genet.

[6] Healy JK (2014). Pseudohypoaldosteronism type II: history, arguments, answers, and still some questions. Hypertension.

[7] Paver WK, Pauline GJ (1964). Hypertension and hyperpotassaemia without renal disease in a young male. Med J Aust.

[8] Stokes GS, Gentle JL, Edwards KD, Stewart JH (1968). Syndrome of idiopathic hyperkalaemia and hypertension with decreased plasma renin activity: effects on plasma renin and aldosterone of reducing the serum potassium level. Med J Aust.

[9] Arnold JE, Healy JK (1969). Hyperkalemia, hypertension and systemic acidosis without renal failure associated with a tubular defect in potassium excretion. Am J Med.

[10] Gordon RD, Geddes RA, Pawsey CG, O'Halloran MW (1970). Hypertension and severe hyperkalaemia associated with suppression of renin and aldosterone and completely reversed by dietary sodium restriction. Australas Ann Med.

[11] Wilson FH, Disse-Nicodeme S, Choate KA, Ishikawa K, Nelson-Williams C, Desitter I (2001). Human hypertension caused by mutations in WNK kinases. Science.

[12] Boyden LM, Choi M, Choate KA, Nelson-Williams CJ, Farhi A, Toka HR (2012). Mutations in kelch-like 3 and cullin 3 cause hypertension and electrolyte abnormalities. Nature.

[13] Louis-Dit-Picard H, Barc J, Trujillano D, Miserey-Lenkei S, Bouatia-Naji N, Pylypenko O (2012). KLHL3 mutations cause familial hyperkalemic hypertension by impairing ion transport in the distal nephron. Nat Genet.

[14] Kliuk-Ben Bassat O, Carmon V, Hanukoglu A, Ganon L, Massalha E, Holtzman EJ (2017). Familial hyperkalemia and hypertension (FHHt) and KLHL3: description of a family with a new recessive mutation (S553L) compared to a family with a dominant mutation, Q309R, with analysis of urinary sodium chloride cotransporter. Nephron.

[15] Zhang R, Zhang S, Luo Y, Li M, Wen X, Cai X (2021). A case report of pseudohypoaldosteronism type II with a homozygous KLHL3 variant accompanied by hyperthyroidism. BMC Endocr Disord.

[16] Farfel Z, Iaina A, Rosenthal T, Waks U, Shibolet S, Gafni J (1978). Familial hyperpotassemia and hypertension accompanied by normal plasma aldosterone levels: possible hereditary cell membrane defect. Arch Intern Med.

[17] Castaneda-Bueno M, Cervantes-Perez LG, Vazquez N, Uribe N, Kantesaria S, Morla L (2012). Activation of the renal Na+:Cl-cotransporter by angiotensin II is a WNK4-dependent process. Proc Natl Acad Sci U S A.

[18] Sohara E, Uchida S (2016). Kelch-like 3/cullin 3 ubiquitin ligase complex and WNK signaling in salt-sensitive hypertension and electrolyte disorder. Nephrol Dial Transplant.

[19] Shibata S, Zhang J, Puthumana J, Stone KL, Lifton RP (2013). Kelch-like 3 and cullin 3 regulate electrolyte homeostasis via ubiquitination and degradation of WNK4. Proc Natl Acad Sci U S A.

[20] Kahle KT, Wilson FH, Leng Q, Lalioti MD, O'Connell AD, Dong K (2003). WNK4 regulates the balance between renal NaCl reabsorption and K+ secretion. Nat Genet.

[21] Ring AM, Cheng SX, Leng Q, Kahle KT, Rinehart J, Lalioti MD (2007). WNK4 regulates activity of the epithelial Na+ channel in vitro and in vivo. Proc Natl Acad Sci U S A.

[22] Sasaki E, Susa K, Mori T, Isobe K, Araki Y, Inoue Y (2017). KLHL3 knockout mice reveal the physiological role of KLHL3 and the pathophysiology of pseudohypoaldosteronism type II caused by mutant KLHL3. Mol Cell Biol.

[23] Dhanoa BS, Cogliati T, Satish AG, Bruford EA, Friedman JS (2013). Update on the Kelch-like (KLHL) gene family. Hum Genomics.

[24] Waterhouse AM, Procter JB, Martin DM, Clamp M, Barton GJ (2009). Jalview version 2: a multiple sequence alignment editor and analysis workbench. Bioinformatics.

[25] Ren J, Wen L, Gao X, Jin C, Xue Y, Yao X (2009). DOG 1.0: illustrator of protein domain structures. Cell Res.

[26] Schumacher FR, Sorrell FJ, Alessi DR, Bullock AN, Kurz T (2014). Structural and biochemical characterization of the KLHL3-WNK kinase interaction important in blood pressure regulation. Biochem J.

[27] Humphrey W, Dalke A, Schulten K (1996). VMD: visual molecular dynamics. J Mol Graph.

[28] Webb B, Sali A (2016). Comparative protein structure modeling using MODELLER. Curr Protoc Bioinformatics.

[29] Olsson MH, Søndergaard CR, Rostkowski M, Jensen JH (2011). PROPKA3: consistent treatment of internal and surface residues in empirical pKa predictions. J Chem Theory Computat.

[30] Abraham MJ, Murtola T, Schulz R, Páll S, Smith JC, Hess B (2015). GROMACS: high performance molecular simulations through multi-level parallelism from laptops to supercomputers. SoftwareX.

[31] Huang J, Rauscher S, Nawrocki G, Ran T, Feig M, de Groot BL (2017). CHARMM36m: an improved force field for folded and intrinsically disordered proteins. Nat Methods.

[32] Won Y (2012). Force field for monovalent, divalent, and trivalent cations developed under the solvent boundary potential. J Phys Chem A.

[33] Hess B, Bekker H, Berendsen HJC, Fraaije JGEM (1997). LINCS: a linear constraint solver for molecular simulations. J Comput Chem.

[34] Essmann U, Lalith P, Berkowitz ML, Darden T, Lee L, Pedersen LG (1995). A smooth particle mesh Ewald method. J Chem Phys.

[35] Bussi G, Donadio D, Parrinello M (2007). Canonical sampling through velocity rescaling. J Chem Phys.

[36] Berendsen HJC, Postma JPM, van Gunsteren WF, DiNola A, Haak JR (1984). Molecular dynamics with coupling to an external bath. J Chem Phys.

[37] Parinello MRA (1981). Polymorphic transitions in single crystals: a new molecular dynamics method. J Appl Phys.

[38] Pettersen EF, Goddard TD, Huang CC, Meng EC, Couch GS, Croll TI (2021). UCSF ChimeraX: structure visualization for researchers, educators, and developers. Protein Sci.

[39] Kostritskii AY, Alleva C, Cönen S, Machtens JP (2021). g_elpot: a tool for quantifying biomolecular electrostatics from molecular dynamics trajectories. J Chem Theory Comput.

[40] Levey AS, Stevens LA, Schmid CH, Zhang YL, Castro AF, Feldman HI (2009). A new equation to estimate glomerular filtration rate. Ann Intern Med.

[41] Adzhubei IA, Schmidt S, Peshkin L, Ramensky VE, Gerasimova A, Bork P (2010). A method and server for predicting damaging missense mutations. Nat Methods.

[42] Ng PC, Henikoff S (2003). SIFT: predicting amino acid changes that affect protein function. Nucleic Acids Res.

[43] Schwarz JM, Cooper DN, Schuelke M, Seelow D (2014). MutationTaster2: mutation prediction for the deep-sequencing age. Nat Methods.

[44] Karczewski KJ, Francioli LC, Tiao G, Cummings BB, Alfoldi J, Wang Q (2020). The mutational constraint spectrum quantified from variation in 141,456 humans. Nature.

[45] Li X, Zhang D, Hannink M, Beamer LJ (2004). Crystal structure of the Kelch domain of human Keap1. J Biol Chem.

[46] Wang L, Jiang C, Cai R, Chen XZ, Peng JB (2019). Unveiling the distinct mechanisms by which disease-causing mutations in the Kelch domain of KLHL3 disrupt the interaction with the acidic motif of WNK4 through molecular dynamics simulation. Biochemistry.

[47] Wakabayashi M, Mori T, Isobe K, Sohara E, Susa K, Araki Y (2013). Impaired KLHL3-mediated ubiquitination of WNK4 causes human hypertension. Cell Rep.

[48] Seifarth C, Trenkel S, Schobel H, Hahn EG, Hensen J (2002). Influence of antihypertensive medication on aldosterone and renin concentration in the differential diagnosis of essential hypertension and primary aldosteronism. Clin Endocrinol.

[49] Stowasser M, Ahmed AH, Pimenta E, Taylor PJ, Gordon RD (2012). Factors affecting the aldosterone/renin ratio. Horm Metab Res.

[50] Stratton JD, McNicholas TA, Farrington K (1998). Recurrent calcium stones in Gordon's syndrome. Br J Urol.

[51] Mayan H, Munter G, Shaharabany M, Mouallem M, Pauzner R, Holtzman EJ (2004). Hypercalciuria in familial hyperkalemia and hypertension accompanies hyperkalemia and precedes hypertension: description of a large family with the Q565E WNK4 mutation. J Clin Endocrinol Metab.

[52] Mori Y, Wakabayashi M, Mori T, Araki Y, Sohara E, Rai T (2013). Decrease of WNK4 ubiquitination by disease-causing mutations of KLHL3 through different molecular mechanisms. Biochem Biophys Res Commun.

