# Supplementary material for: A Novel Homozygous KLHL3 Mutation as a Cause of Autosomal Recessive Pseudohypoaldosteronism Type II Diagnosed Late in Life
Source: Nephron. 2022 Jan 28;146(4):418–28. doi: 10.1159/000521626 (PMC9393831; doi:10.1159/000521626)
Supplement: Supplementary file 1 — Supplementary data [file nef-0146-0418-s01.docx]

**Online Supplementary Material**

**Supplemental Table 1**

**Paralogs used for alignment**

| **Gene name** | **Protein name** | **NCBI Reference Sequence ID** |
| --- | --- | --- |
| KLHL1 | Kelch-like protein 1 isoform 1 | NP_065917.1 |
| KLHL2 | Kelch-like protein 2 isoform 1 | NP_009177.3 |
| KLHL3 | Kelch-like protein 3 isoform 1 | NP_059111.2 |
| KLHL4 | Kelch-like protein 4 isoform 1 | NP_061990.2 |
| KLHL5 | Kelch-like protein 5 isoform 1 | NP_057074 |
| KLHL6 | Kelch-like protein 6 | NP_569713.2 |
| KLHL7 | Kelch-like protein 7 isoform 1 | NP_001026880.2 |
| KLHL8 | Kelch-like protein 8 isoform 1 | NP_001278932.1 |
| KLHL9 | Kelch-like protein 9 | NP_061335.1 |
| KLHL10 | Kelch-like protein 10 isoform 1 | NP_001316524.1 |
| KLHL11 | Kelch-like protein 11 precursor | NP_060613.1 |
| KLHL12 | Kelch-like protein 12 isoform 1 | NP_001289980.1 |
| KLHL13 | Kelch-like protein 13 isoform a | NP_277030.2 |
| KLHL14 | Kelch-like protein 14 | NP_065856.1 |
| KLHL15 | Kelch-like protein 15 | NP_085127.2 |
| GAN1 (KLHL16) | Gigaxonin isoform 1 | NP_071324.1 |
| KLHL17 | Kelch-like protein 17 | NP_938073.1 |
| KLHL18 | Kelch-like protein 18 | NP_079286.2 |
| KEAP1 (KLHL19) | Kelch-like ECH-associated protein 1 | NP_036421.2 |
| KLHL20 | Kelch-like protein 20 | NP_055273.2 |
| KLHL21 | Kelch-like protein 21 isoform 1 | NP_055666.2 |
| KLHL22 | Kelch-like protein 22 | NP_116164.2 |
| KLHL23 | Kelch-like protein 23 | NP_001186219.1 |
| KLHL24 | Kelch-like protein 24 isoform a | NP_001336342.1 |
| KLHL25 | Kelch-like protein 25 | NP_071925.2 |
| KLHL26 | Kelch-like protein 26 | NP_001332910.1 |
| IPP (KLHL27) | Actin-binding protein IPP isoform 1 | NP_005888.1 |
| KLHL28 | Kelch-like protein 28 isoform 1 | NP_001295041.1 |
| KLHL29 | Kelch-like protein 29 | NP_443152.1 |
| KLHL30 | Kelch-like protein 30 | NP_940984.3 |
| KLHL31 | Kelch-like protein 31 | NP_940984.3 |
| KLHL32 | Kelch-like protein 32 isoform a | NP_001310181.1 |
| KLHL33 | Kelch-like protein 33 isoform 1 | NP_001352719.1 |
| KLHL34 | Kelch-like protein 34 | NP_695002.1 |
| KLHL35 | Kelch-like protein 35 | NP_001034637.2 |
| KLHL36 | Kelch-like protein 36 isoform 1 | NP_079007.2 |
| ENC1 (KLHL37) | Ectoderm-neural cortex protein 1 isoform 1 | NP_001243503.1 |
| KLHL38 | Kelch-like protein 38 | NP_001075144.2 |
| NS1BP (KLHL39) | Influenza virus NS1A-binding protein | NP_006460.2 |
| KLHL40 | Kelch-like protein 40 | NP_689606.2 |
| KLHL41 | Kelch-like protein 41 | NP_006054.2 |
| KLHL42 | Kelch-like protein 42 | NP_065833.1 |

**Supplemental Figure 1**

**
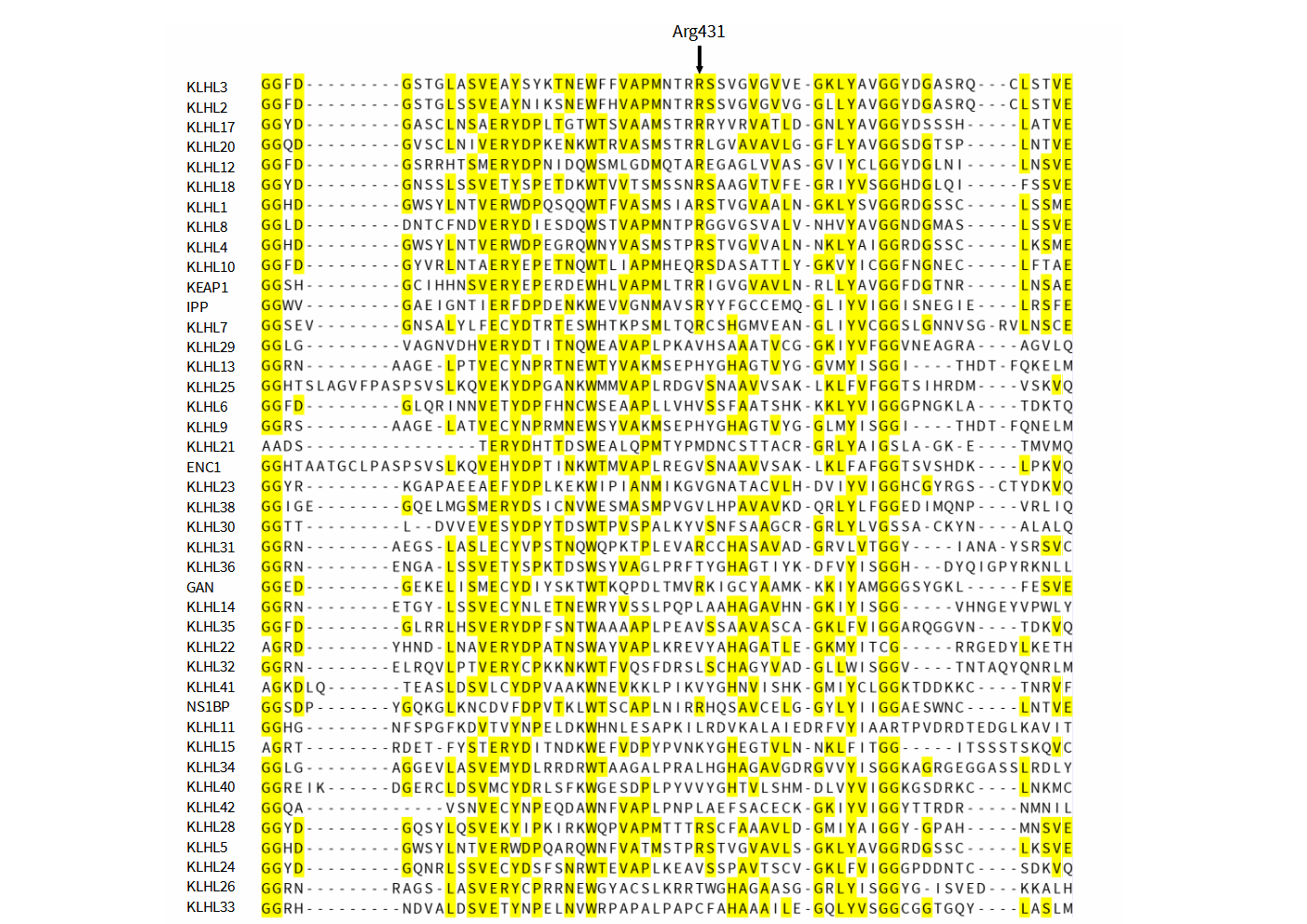
**

Alignment of human paralogs demonstrates partial conservation of Arg431. None of the paralogs has tryptophan (W) at the homologous position.
